# Equine osteoarthritis: Strategies to enhance mesenchymal stromal cell-based acellular therapies

**DOI:** 10.3389/fvets.2023.1115774

**Published:** 2023-02-10

**Authors:** Manon Jammes, Romain Contentin, Frédéric Cassé, Philippe Galéra

**Affiliations:** BIOTARGEN, UNICAEN, Normandie University, Caen, France

**Keywords:** osteoarthritis, mesenchymal stromal cells, extracellular vesicles, acellular therapy, horse

## Abstract

Osteoarthritis (OA) is a degenerative disease that eventually leads to the complete degradation of articular cartilage. Articular cartilage has limited intrinsic capacity for self-repair and, to date, there is no curative treatment for OA. Humans and horses have a similar articular cartilage and OA etiology. Thus, in the context of a One Health approach, progress in the treatment of equine OA can help improve horse health and can also constitute preclinical studies for human medicine. Furthermore, equine OA affects horse welfare and leads to significant financial losses in the equine industry. In the last few years, the immunomodulatory and cartilage regenerative potentials of mesenchymal stromal cells (MSCs) have been demonstrated, but have also raised several concerns. However, most of MSC therapeutic properties are contained in their secretome, particularly in their extracellular vesicles (EVs), a promising avenue for acellular therapy. From tissue origin to *in vitro* culture methods, various aspects must be taken into consideration to optimize MSC secretome potential for OA treatment. Immunomodulatory and regenerative properties of MSCs can also be enhanced by recreating a pro-inflammatory environment to mimic an *in vivo* pathological setting, but more unusual methods also deserve to be investigated. Altogether, these strategies hold substantial potential for the development of MSC secretome-based therapies suitable for OA management. The aim of this mini review is to survey the most recent advances on MSC secretome research with regard to equine OA.

## 1. Introduction

Articular cartilage is the connective tissue that covers the extremities of bones in diarthrodial joints. Its viscoelasticity allows for shock absorption and joint mobility without friction (1). Articular cartilage is composed of specialized cells called chondrocytes, and an abundant extracellular matrix mainly enriched in type II collagen (Col II) and aggrecan. Osteoarthritis (OA) is a degenerative joint disease that, in its later stages, affects the whole joint and leads to decreased joint mobility, pain and impaired quality of life. During OA, articular cartilage homeostasis is disrupted and the overproduction of catabolic enzymes, such as matrix metalloproteinases (MMP) and aggrecanases, leads to cartilage degradation, articular inflammation and, eventually, subchondral bone exposure (1). OA management is challenging because cartilage has a limited capacity for self-repair. To date, there are no curative OA treatments.

As in humans, horses can develop OA due to aging or intense physical activity, directly affecting horse health and welfare, and diminishing performance in sport and race horses (2). OA can thus put an early end to a horse career, leading to economic losses (3). The horse is also an excellent preclinical model for OA, because human and equine articular cartilage share many similarities in terms of anatomy, mechanical functioning, and cellular and molecular composition (2, 4).

Current clinical treatments of equine OA such as anti-inflammatory drugs, dietary supplements or viscosupplementation are only symptomatic and do not prevent the degenerative process of the disease (2). However, among the various emerging regenerative therapies, strategies based on mesenchymal stromal cells (MSCs) appear to hold promise. MSCs possess immunomodulatory and anti-inflammatory effects and regenerative properties that have direct effects or act indirectly through the release of bioactive molecules free or enclosed in extracellular vesicles (EVs) such as exosomes or ectosomes (5).

Here, we explore the relevance and future challenges of MSC-derived EVs (MSC-EVs) as a new orthobiologic approach to manage equine OA.

## 2. Mesenchymal stromal cell-based therapies

MSCs are defined as multipotent cells able to self-replicate and differentiate into distinct cell types, such as adipocytes, osteoblasts or chondrocytes. Bone marrow (BM) is the most common source of MSCs, even though these cells can be found in several other niches in an organism [adipose tissue (AT), umbilical cord (UC), dental pulp, synovium, etc.] (5). Research over the last few years has suggested that MSCs hold great potential for diverse therapeutic applications through putative immunomodulatory, anti-inflammatory effects or by stimulating tissue regeneration (6–9).

Regarding articular diseases, MSCs have shown the potency to reduce OA-related pain and increase cartilage repair (6, 9). Additionally, OA-afflicted horses treated with intra-articular injections of MSCs show improvement in clinical signs, cartilage appearance and athletic performance (10–12). In the context of autologous chondrocyte transplantation (ACT), equine MSC (eMSC)-derived cartilage organoids overcome the limitations inherent to the use of dedifferentiated chondrocytes and may provide an accurate and reliable drug screening model for OA (13–17).

**Figure 1:**
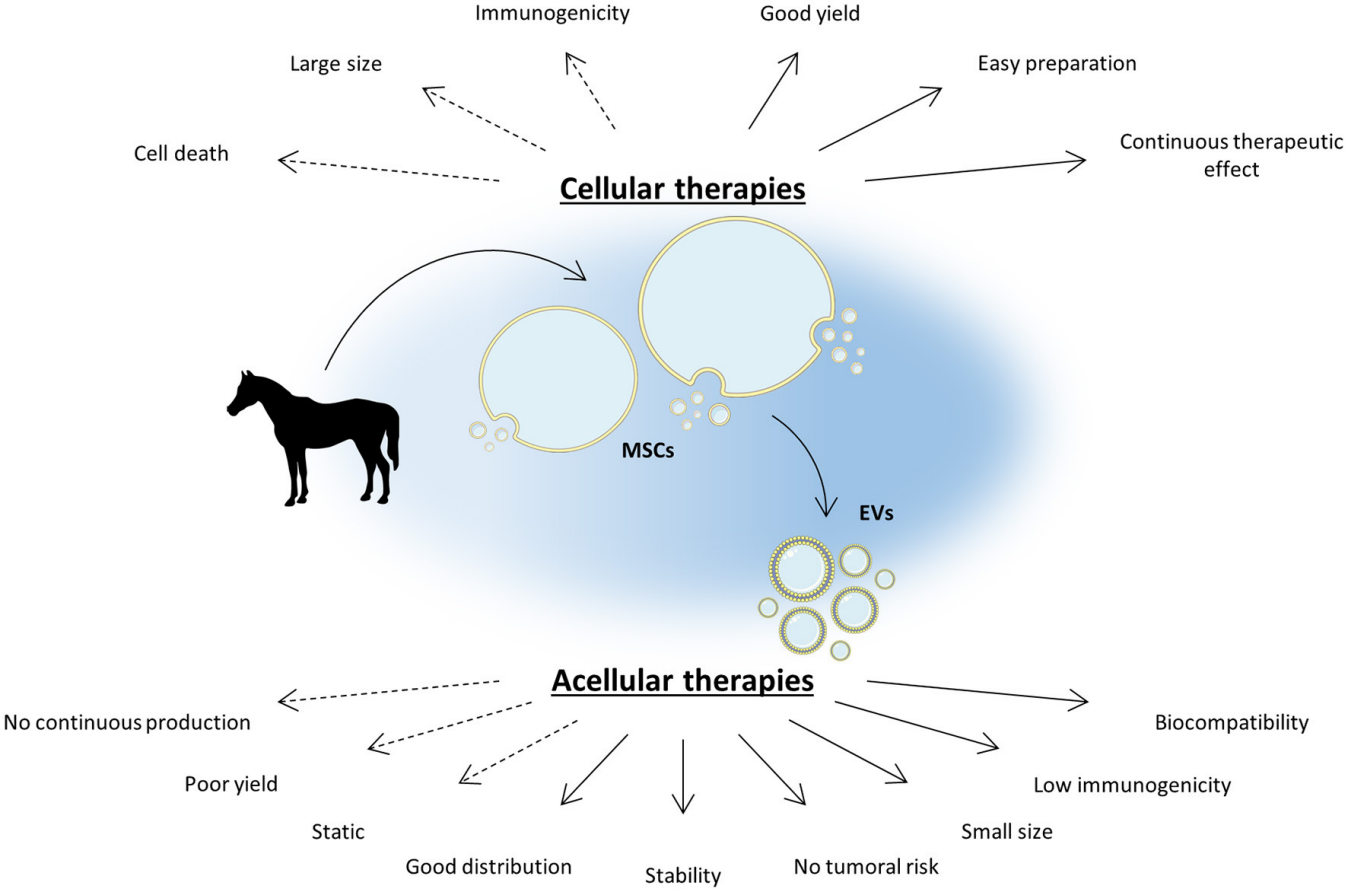
MSC-based acellular therapies are an appealing strategy to bypass cellular therapy limitations. Cellular therapies based on mesenchymal stromal cells (MSCs) demonstrate a promising potential in the management of equine osteoarthritis (OA) by reducing joint inflammation and enhancing cartilage regeneration. However, MSC intra-articular injection can be immunogenic and brings several concerns inherent to their nature. As the main part of MSC therapeutic potential lies in their secretome, notably in extracellular vesicles (EVs), acellular therapies appear to be a promising alternative to increase the safety of MSC-based therapy while improving its efficacy. EVs are highly biocompatible and can easily diffuse into tissues thanks to their small size. To date, acellular therapies still exhibit a few limitations but several strategies are in progress to overcome them. The Figure was partly generated using Servier Medical Art, provided by Servier, licensed under a Creative Commons Attribution 3.0 unported license.

Although the direct use of MSCs remains promising for equine OA treatments, several challenges have been identified, including their *in vivo* distribution, a low engraftment rate, their immunogenicity and their possible tumorigenicity risk (18–24) (Figure 1).

## 3. Mesenchymal stromal cell-derived extracellular vesicles as a new orthobiologic therapy

The MSC secretome contains a broad spectrum of compounds including nucleic acids, proteins such as cytokines, growth factors or even lipids. Some of these compounds can be encapsulated in vesicles, called EVs. EVs include apoptotic bodies (>1 μm diameter), ectosomes or microvesicles (100–1,000 nm) and exosomes (30–200 nm). Among the MSC secretome, exosomes contain numerous molecules with proven pro-regenerative and anti-inflammatory properties as reviewed in Hade et al. (25). In addition, numerous studies have demonstrated the cartilage regeneration potential of EVs (26).

Exosomes originate from the endocytic pathway, develop within multivesicular bodies (MVBs) and are delivered to the extracellular environment when MVBs fuse with the plasma membrane (27). Exosomes enter the cells through membrane fusion, endocytosis or interaction with a receptor that is subsequently internalized.

Given the disadvantages attributed to MSC-based cellular therapy, secretome-, EV- and exosome-based strategies are an appealing alternative to explore the therapeutic potential of eMSCs in equine OA management. To date, only a few studies have demonstrated the therapeutic potential of eMSC-EVs in the context of horse OA. Using an *in vitro* cartilage organoid model, our research group has already demonstrated the pro-anabolic potential of eMSC-conditioned media (CM) and the presence of exosomes in eMSC-CM (28). The CM corresponds to the medium in which cell were cultured, hence it contains components that had been secreted by the cells. Noteworthy, because cells cultured *in vitro* do not have exactly the same features than their *in vivo* counterparts, the CM and the secretome of MSC *in vivo* might differ. MSC-EVs can decrease the transcript levels of MMP and pro-inflammatory molecules (29, 30). Furthermore, EVs can be used as biomarkers to evaluate the progression of OA (31). However, to date, *in vivo* cartilage regeneration using EVs in the equine model remains to be demonstrated.

Nevertheless, environment deeply influences MSC secretion and constitutes a variable worth of consideration to improve the capacities attributed to their therapeutic effect (32–34). The MSC therapeutic potential and secretome differ according to the tissue they derive from and can be modulated by several factors as discussed below.

## 4. Therapeutic potential of mesenchymal stromal cells and their derivatives depends on the source and the culture procedure

MSCs from all sources share similarities, regarding in particular their self-renewal, multipotency and immunomodulation capacities. Nevertheless, the individual, the age, the tissue and the niche MSCs are isolated from lead to slight variations of their properties (35) including their secretory production. For example, AT-eMSCs, peripheral blood (PB)-eMSCs, BM-eMSCs and UC-eMSCs display significant variation in inflammation-related gene expression, although interferon-γ (IFN-γ) stimulation homogenizes the gene expression profile between the studied MSC sources (36). Furthermore, their immunomodulatory properties can be induced through different mechanisms. For example, AT-eMSCs and UC-eMSCs can induce lymphocyte apoptosis, whereas BM-eMSCs, PB-eMSCs and cord blood (CB)-eMSCs induce lymphocyte cell cycle arrest (37). Our research group has demonstrated differences in proliferation and differentiation capacity between BM-eMSCs, CB-eMSCs and UC-eMSCs (13–17). BM-eMSCs are more prone to produce hyaline-like cartilage extracellular matrix (ECM) with low amounts of atypical molecules than are CB-eMSCs and UC-eMSCs. The impact of eMSC origin on antibacterial activity has also been demonstrated (38) and the eMSC secretome also depends on the tissue source they derive from. Indeed, Navarette et al. have reported that the miRNA content of EVs differs between AT- and endometrial eMSCs from the same animal (39). In addition to the inter-tissue origin heterogeneity, eMSCs derived from the same tissue source can show differences in gene expression and functional heterogeneity (40). Ultimately, the differentiation status of MSCs modulates their properties. As an example, BM-eMSCs engaged in a chondrogenic differentiation process exert a weaker inflammatory response to IL-1β than naive BM-eMSCs (41). eMSC heterogeneity suggests that these cells are highly influenced by their environment. Considering the impact of MSC origin on their properties, tissue source should be wisely selected before exploiting their secretome. Hence, in the study of the therapeutic potential of eMSCs, medium composition and culture conditions must be carefully selected.

Culture medium supplementation with fetal bovine serum (FBS) is widely used to support *in vitro* MSC proliferation. One of the challenges in the use of FBS resides in its non-standardized and variable composition between batches (42). This xenogeneic supplement can interfere with MSC metabolism, phenotype and, by consequence, the properties of their secretome. Additionally, FBS is a limiting factor in *in vivo* applications because it can trigger an immune response. For instance, eMSCs cultured with FBS have exacerbated immunogenicity compared with eMSCs cultured with allogenic or autogenic equine BM supernatant-supplemented culture medium (43). The use of autologous equine serum can be considered for the culturing eMSCs because horses can tolerate the removal of 25% of their blood volume (44). Replacing FBS with equine platelet lysate as a medium supplement has also been tested, and resulted in similar growth and phenotypical BM-MSC characteristics (45) as well in moderately increased immunomodulatory marker expression (46). In contrast, Pezzanite et al. demonstrated the superiority of FBS over equine serum supplementation to generate functional eMSCs (47). Serum-free medium is another alternative to FBS supplementation during MSC expansion. This option is being investigated, especially during CM and EV harvest, to avoid the co-isolation of xenogeneic contaminants that can reduce the therapeutic efficacy of EVs. Serum-free-cultured eMSCs decrease the pro-inflammatory mediator secretion of activated T-cells, but to a lesser extent than eMSCs cultured with FBS (48). In the last few decades, efforts have been made to culture the cells *in vitro* in contexts similar to those *in vivo*, particularly using three-dimensional (3D) cultures instead of monolayers (2D). MSCs cultured in 3D undergo morphological and metabolic changes, and their proliferation and survival rate are increased (49). Compared with a monolayer culture, CM from MSCs cultured in spheroids suppress macrophage pro-inflammatory cytokine secretion and enhance the production of the anti-inflammatory cytokine interleukin (IL)-10 (50). Additionally, MSCs grown in dynamic 3D cultures—spinner flasks and a rotating bioreactor—show enhanced therapeutic properties, but mRNA profiles differ according to the method used (51). To date, these culture methods have not yet been tested in the equine model.

Long-term *in vitro* expansion affects MSCs (52). A large proportion of MSCs become senescent and display altered differentiation and immunosuppressive potential (53, 54). Therefore, early passages should be preferred to harness eMSC therapeutic properties. Noteworthily, cryopreservation does not appear to interfere with eMSC differentiation potential and therapeutic potential (55, 56), but the isolation protocol can affect the characteristics of these cells (57). Nevertheless, the MSC secretome, particularly EVs, can exhibit diminished immunosuppressive properties after freeze-thawing (58).

**Figure 2:**
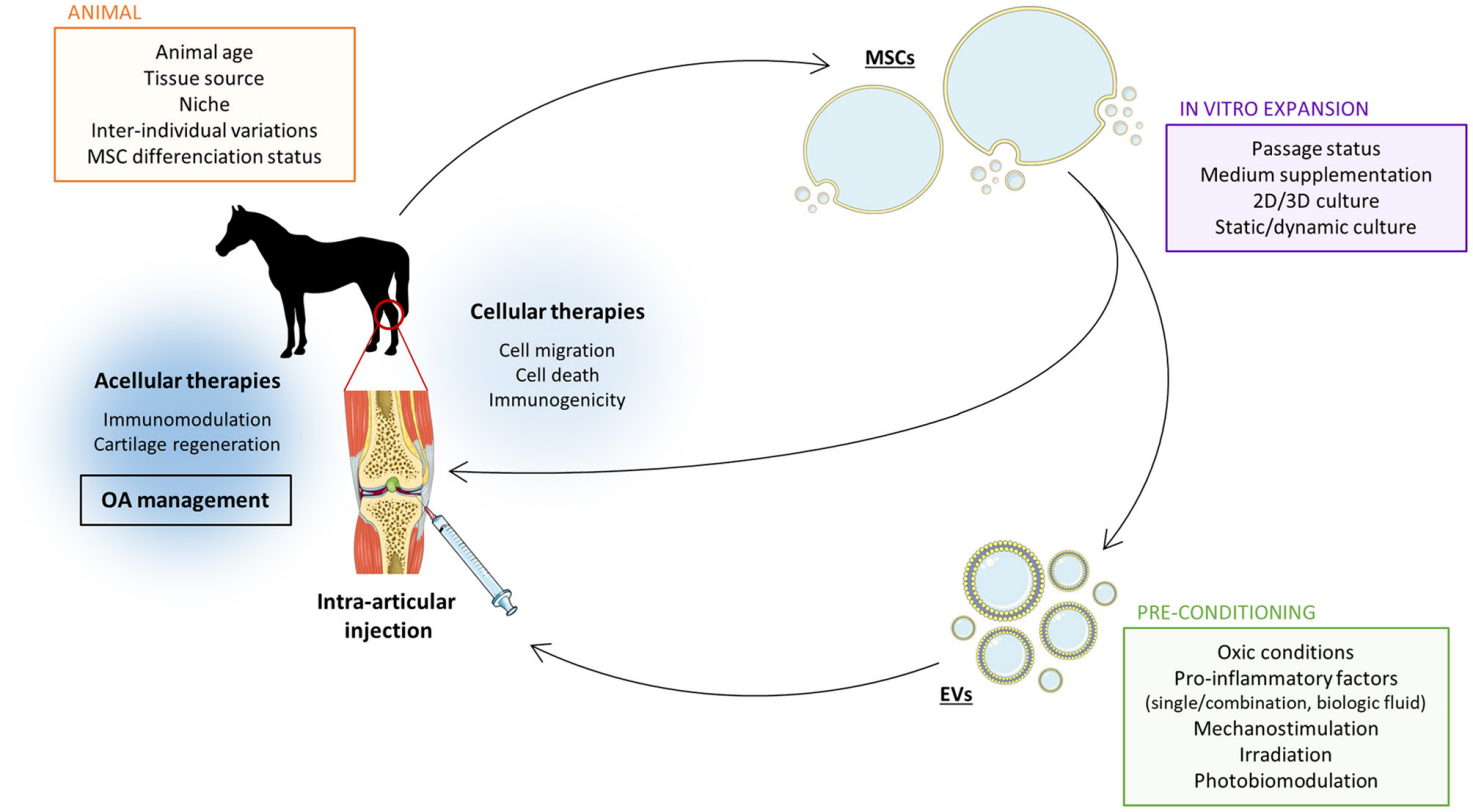
Several parameters must be considered to improve the therapeutic potential of MSC-derived EVs in the treatment of equine OA. Mesenchymal stromal cells (MSC)-derived extracellular vesicles (EVs) have the capacity to improve equine osteoarthritis (OA) management *via* immunomodulation and stimulation of cartilage matrix synthesis. However, this therapeutic potential can be enhanced by adjusting the steps in the EV production protocol. The source of MSCs affects EV properties and their therapeutic capacities. The MSC culture methods can be used as a tool to optimize the therapeutic effect of MSC-derived EVs. In the last years, it has been demonstrated that EV properties can be refined by adjusting oxic conditions, adding pro-inflammatory cytokines or other physical parameters such as mechanical stress or light exposition. Setting the balance between all these factors is crucial to achieve the most effective therapeutic effect of MCS-derived EVs for equine OA. Moreover, acellular therapy has the advantage to avoid most of direct MSC intra-articular injections concerns such as immunogenicity. The Figure was partly generated using Servier Medical Art, provided by Servier, licensed under a Creative Commons Attribution 3.0 unported license.

The surrounding environment inevitably affects eMSCs. Controlling it remains a real challenge that must be addressed to increase the reproducibility of the therapeutic effects of MSCs or MSC-derived products such as EVs. On the other hand, the ability of MSCs to adapt to their environment also represents a tremendous opportunity to improve their therapeutic potential (Figure 2).

## 5. Future directions to enhance the therapeutic potential of MSC and their derivatives

It has already been proven that the unstimulated eMSC secretome can enhance the equine articular chondrocyte phenotype and increase their migratory capacity (28, 30). Nevertheless, therapeutic capacities of naive MSCs developing in a healthy environment have not been optimized. To maximize the MSC therapeutic properties, stimulation by extrinsic factors can mimic a pathological situation, leading to a boost in the MSC immunomodulatory and therapeutic capacities (8). In OA, this is illustrated *in vivo* by a pro-inflammatory environment induced by a cartilage lesion, triggering an MSC reaction to external aggression. These diverse procedures are collectively called preconditioning, licensing or priming and are probably the key to improvements in the regenerative potential of the MSC secretome (Figure 2).

Given that MSCs evolve *in vivo* in hypoxic conditions (2%−9% oxygen) (59), growing them under a 21% oxygen atmosphere can alter their phenotype and their secretome. Low oxygen tension regulates hypoxia-inducible factor (HIF-1α) activity that triggers the transcription of diverse genes involved in eMSC stemness-associated features, differentiation and self-renewal (60). Because EV cargo reflects the nature and composition of their cell source, these factors are likely to be found in the eMSC secretome and modify their properties (61). Recently, Zhang et al. (62) showed that the secretome of hypoxia-preconditioned MSCs enhanced rat chondrocyte proliferation and migration and inhibited apoptosis compared with rat chondrocytes cultured with the secretome from MSCs grown in normoxia. To our knowledge, none of the hypoxia preconditioning advantages described previously have yet been demonstrated in equines.

When tissue is damaged, inflammatory factors and chemokines are released by immune cells recruited to the inflammation site, triggering eMSC activation. Reproducing this process *in vitro* is one way to enhance eMSC-EV therapeutic capacities. Interferon γ (IFN-γ) is known as the gold standard cytokine priming for MSCs. Many studies confirm its abilities to enhance eMSC secretome-mediated chondroprotection and downregulate inflammatory genes in equine chondrocytes (63, 64). IFN-γ can also increase the immunosuppressive properties of murine BM-MSCs, but priming does not enhance the capacities of EVs (58). Depending on their source, eMSCs vary in their response to IFN-γ, but this cytokine lowers inter-tissue differences in unstimulated eMSC immunomodulatory gene expression (36). Therefore, tissue of origin may not be a crucial parameter when IFN-γ is used to license eMSCs. Moreover, eMSC surface expression of major histocompatibility complex (MHC)-II in horses is increased by IFN-γ and decreased by IL-1β. However, tumor necrosis factor-α (TNF-α) has no impact on the expression of MHC-II, demonstrating the importance of the nature of the cytokine used for eMSC stimulation on their antigenicity and immunomodulation (65).

However, a single molecule is not an accurate replication of the whole inflammatory environment. Pro-inflammatory cytokines can exert distinct actions. For example, preconditioning human MSCs with TNF-α enhances the chondrogenic differentiation potential of the cells, whereas IL-1β does not enhance the chondrogenic potential of MSCs (66). Thus, a combination of several of these factors may be more accurate. Stimulation of eMSCs with IFN-γ and TNF-α resulted in the overexpression of immunomodulation-related genes (67). Alone or in combination, these cytokines also significantly increased the expression of prostaglandin-endoperoxide synthase 2 (PGE2) and indoleamine 2,3-dioxygenase (IDO) in eMSCs (68). PGE2 (37) and IDO both mediate most of the inhibition of equine lymphocyte proliferation, although the involvement of IDO in the horse model is a subject of debate (69). Recently, injection of the secretome from TNFα and IFNγ-stimulated eMSCs in LPS-induced osteoarthritic equine joints (70) led to reduced inflammatory symptoms and higher ECM marker expression in joints treated with concentrated CM. Nonetheless, no differences were noted between MSC-secretome and MSC groups. To our knowledge, this is the only MSC-CM *in vivo* experiment that has been carried out in the equine model.

However, combining cytokines only considers a small part of *in vivo* molecular content and interactions. Cytokine priming can have a deleterious effect on eMSC viability and trilineage differentiation, which is not observed when they are primed with inflammatory synovial fluid (SF) (71). Because immunomodulatory cytokines are also released by activated T-cells, CM from PB mononuclear cell-activated eMSCs can diminish T-cell proliferation in a significative manner compared with naive eMSCs (69). Platelet-rich plasma (PRP) and bone marrow concentrate (BMC) can stimulate the migration of eMSCs (72). These biological fluids contain various healing-related factors and, because migration is linked to immunomodulation (73), they may represent a strategy for eMSC preconditioning.

Hypoxia and cytokine priming are the most investigated strategies for improving eMSC therapeutic potential. Nevertheless, some less well-known methods may be promising. eMSCs are naturally exposed to mechanical forces such as fluid shear stress, hydrostatic compression or mechanical loading that affect MSC proliferation, differentiation and migration (74). Moreover, the human MSC secretome's ability to modulate angiogenesis is influenced by the mechanical environment of MSCs in both 2D and 3D culture systems (75). To date, mechanostimulation efficiency has not been demonstrated in the equine model. Extracorporeal shock wave therapy (ESWT) is a type of mechanical sensing using acoustic waves already employed in the therapies for tendon and ligament affections, but only as an auxiliary treatment in equine OA management. ESWT can increase metabolic activity and differentiation of eMSCs, but no effects on immunomodulatory potential have been observed (76). Furthermore, CM from human MSCs exposed to pulsed electromagnetic fields can also enhance articular chondrocyte migration and reduce the inflammatory state and apoptosis of these cells (77), but no proof is available regarding equine cells. Lastly, 1064 nm irradiation enhances IL-10 and VEGF expression in naive eMSCs (78). Photobiomodulation may be another way of maximizing stimulation and therapeutic potential of eMSCs in the treatment of equine OA.

## 6. Conclusion

The relevance of the equine model in OA therapy research contributes to the emergence of new studies and better understanding of the therapeutic potential of eMSCs. Despite recent advances in MSC-based therapies, several hurdles still need be overcome to propose a MSC therapy to treat equine OA. Notwithstanding the difficulties of quantification and large-scale production due to the novelty of the approach, eMSC-EVs may be an appropriate adjunct to improve MSC-based equine OA management. This strategy can benefit from the immunomodulatory, anti-inflammatory and regenerative properties of MSCs without inducing side effects such as immunogenicity or tumoral transformation (Figure 1).

Nonetheless, there still are numerous questions before considering therapies based on MSC-EVs for equine joint disorders. Issues involving eMSC origin, culture and preconditioning conditions, method of EV isolation, enrichment, storage and dosing need to be addressed, as well as the safety of allogenic or autologous EVs (Figure 2). Another critical issue that needs to be examined is the *in vivo* targeting of cartilage. Currently, a promising strategy is the use of a cationic molecule that can coat EVs and reverse their negative surface charge to infiltrate the negatively charged cartilage more easily (79).

To address all these considerations, *in vitro* organoid models of equine chondrocytes or eMSCs can be useful to optimize EV preparation and to identify the ideal treatment for use in controlled clinical trials on horses affected by OA.

Finally, progress in equine OA treatment using the therapeutic potential of MSC-EVs is critical for horse welfare and the equine industry, and may even eventually be transposable to humans as part of a One Health approach.

## Author contributions

Conceptualization, literature search, and writing: MJ, RC, and FC. Review and editing: MJ, RC, FC, and PG. Supervision and approval: PG. All authors contributed to this review and approved the submitted version.
